# Selected Cases Hospital Reports

**Published:** 1880-07

**Authors:** F. Walton


### SELECTED CASES.

COMPILED FROM THE REPORTS OF COLUMBIA VETERINARY COLLEGE HOSPITAL.

BY F. WALTON, D. V. S.

---

CASE I.—Sorrel Gelding. Brought to the Hospital April 7th, with the following history:

The week previous the animal had become suddenly lame in the near hind leg. Warm water, bathing and stimulating liniments were used for three days, when lameness developed in the near forward leg. Warm water and vinegar were now applied, and the following morning the animal was unable to move, being stiff in all its joints. In the evening the horse was brought to the hospital in an ambulance.

On examination, respiration was found accelerated, temperature 103°, pulse 50°, and weak, bowels sluggish, urine scanty and high colored.

Diagnosis—Acute articular rheumatism.

| | | |
|---|---|---|
| Treatment— | Sulphate of magnesia, | 10 oz. |
| | Tr. opium, . . . | 1 oz. |
| | Water, . . . . . | 1 qt. |

D.ench to be administered immediately.

April 8. Apparently less pain. Symptoms unchanged. Ordered salicine in three drachm doses, every two hours.

April 9. Animal much improved. Temperature 100°, pulse normal, but weak. Animal able to lie down.

Continued the salicine in two drachm doses every four hours.

April 10. Salicine in drachm doses every four hours.

April 13. Discharged cured.

CASE II.—Was called to see a bay gelding, age six. Obtained the following history:

For some time groom had noticed that the horse, after any violent exercise, on returning to the stable would manifest symptoms of colic. Recently these symptoms had been more violent.

When I first saw the patient he was straining and making ineffectual efforts to urinate, and was apparently in considerable pain. Occasionally a small amount of urine wou'd escape. Rectal examina'ion disclosed the bladder distended with urine. On attempting to pass the catheter an obstruction was met about two inches below the anus. Gentle manipu'ations succeeded in breaking down a portion of the obstruction, and on withdrawing the instrument it was found filled with very friable calculous deposit. A second attempt resulted in breaking down the entire mass and in successfully introducing the catheter and evacuating the bladder. Evidences of cystic inflammation being found, the blad er was injected with the following:

| | |
|---|---|
| Fluid extract opium, . . . . . | ½ oz. |
| Hydrastis canadensis, . . . . . . | 1 oz. |
| Bicarbonate soda, . . . . . . | 2 drachms. |
| Warm water, . . . . . . . . | 2 qts. |

The treatment continued four days. Animal disc'arged cured.

CASE III.—Bay Gelding. Large heavy animal, at work before a coal cart. Entered the hospital at 8 P. M., May 8th, with the following symptoms: Respirations about 50 per minute, very difficult, and accompanied with a hissing, noisy sonnd. Pulse small and rapid. Temperature 105°. The inspiratory longer than the expiratory act. Schneiderian membranes hot, dry and congested; nostrils dilated, eyes prominent, and nose extended; cold sweat all over the body, legs and ears cold. Animal apparently on the point of suffocation.

Diagnosis—Pharyngo-laryngitis complicated with œdema glottidis.

Treatment—Tracheotomy was at once indicated, but a second thought prompted the following:

Placed the animal in a loose stall, and compelled him to inhale the hot vapor from a pail of hot water in which had been thrown a small quantity of gum camphor. Heated bricks were thrown into similar pails of water placed in different portions of the stall, so that the air of the whole place was kept constantly moist, and at a temperature a little above that of the body of the patient. External applications of hot water were kept constantly applied to the throat and larynx, and the fauces washed out frequently with hot water and chlorate of potass., 1 oz., aq. 2 pts.

In thirty minutes the patient manifested symptoms of relief. In an hour the respirations were reduced to 35 per minute. There was much less whistling and difficulty in breathing. The patient was still sweating profusely, and there was free secretion, with discharge of mucus from the nostrils.

At 10 P.M. the pulse was 50, respiration 20 and quiet, temperature 101°.

Continued hot vapor, and ordered the following liniment to be rubbed over the throat:

R.—Oil of turpentine,
Spirits ammonia,
Olive oil—equal parts.

Applied stimulating liniment and continued the hot vapor.

May 9th. Discontinued vapor; patient doing well; temperature 100°; respiration normal; pulse weak, a little more rapid than in health; slight cough and hoarseness, for the relief of which the following mixture was ordered:

Ext. liquorice Fl.
Ext. senega Fl.
Ext. scilla Fl.
Muriate of ammonia aa, . . . . . 1 oz.
Aqua Pure, . . . . . . . . 8 ozs.

To be repeated every four hours.

Forty-eight hours after time of admission patient was discharged fit for work.

